# An Overview of the Genus *Cotoneaster* (Rosaceae): Phytochemistry, Biological Activity, and Toxicology

**DOI:** 10.3390/antiox9101002

**Published:** 2020-10-16

**Authors:** Agnieszka Kicel

**Affiliations:** Department of Pharmacognosy, Faculty of Pharmacy, Medical University of Lodz, 1 Muszynskiego, 90-151 Lodz, Poland; agnieszka.kicel@umed.lodz.pl

**Keywords:** *Cotoneaster* Medik., ethnobotany, phytochemistry, traditional uses, pharmacology, toxicology

## Abstract

Traditional herbal medicines have become a subject of global importance with both medical and economic implications. The regular consumption of herbal drugs has led to serious concerns regarding their quality, effectiveness, and safety. Thus, relevant scientific evidence has become an important criterion for the acceptance of traditional health claims. The genus *Cotoneaster* Medikus provides numerous species traditionally used in Asian medicine for the treatment of haemorrhoids, diabetes, and cardiovascular diseases. This review summarises the achievements of modern research on the *Cotoneaster* taxa, including ethnobotany, phytochemistry, pharmacology, and toxicology. To date, more than 90 compounds have been isolated or analytically identified in *Cotoneaster* leaves, fruits, flowers or twigs. These phytochemicals are categorised into flavonoids, procyanidins, phenolic acids, cotonefurans, cyanogenic glycosides, triterpenes, sterols, fatty acids, volatile compounds, and carbohydrates, and many of them are responsible for *Cotoneaster* pharmacological properties including antioxidant, anti-inflammatory, antimicrobial, antiparasitic, hepatoprotective, anti-diabetic or anti-dyslipidaemic activity. In order to ensure the safety of pharmaceutical applications, the potential toxicity of *Cotoneaster* extracts has also been investigated. In conclusion, this systematic review provides an important reference base for further study into the various medical applications of both the dry extracts and pure isolates of *Cotoneaster* species.

## 1. Introduction

Herbal plants, used since ancient times in virtually all cultures as a source of medicines, are of great importance to the health of individuals and communities. Traditional medicine is used in all parts of the world and has a rapidly growing economic importance, mainly due to use of both medicinal plants and their standardised dry extracts. The medicinal use of plants of the family Rosaceae is widely described in the scientific literature [1,2]. This family, distributed across approximately 100 genera with almost 3100 species, is one of the most economically important families, comprised of fruit, nut, ornamental, aromatic, herbaceous, and woody plants. Edible Rosaceae crops domesticated for human consumption include apple, strawberry, pear, peach, plum, almond, raspberry, sour cherry, and sweet cherry. Other species, with ornamental and medicinal value, are rose, hawthorn, cotoneaster, potentilla, and pyracantha.

**Figure 1:**
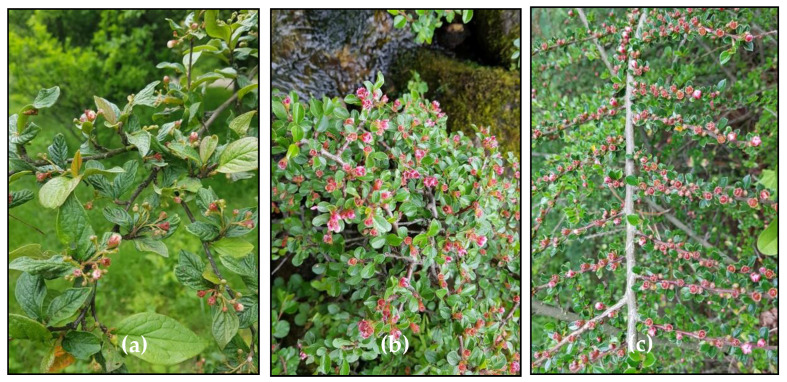
Three representative *Cotoneaster* species: *C. bullatus* (**a**), *C. nanshan* (**b**) and *C. horizontalis* (**c**).

**Figure 2:**
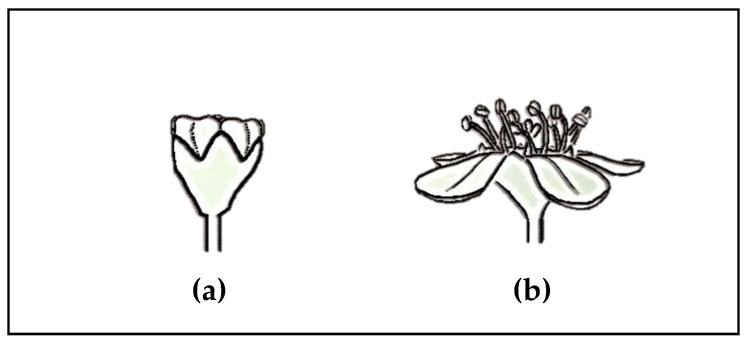
The flower type characteristic of two subgenera: *Cotoneaster* (**a**), and *Chaenopetalum* (**b**) (adapted from [4]).

*Cotoneaster* Medikus, one of the representatives of the Rosaceae family, comprises about 500 species with a Eurasian distribution; the centre of their diversity is in the mountains of China and the Himalayas [3]. This genus, comprising mainly shrubs and small trees, provides popular ornamental plants that are widely grown in landscape architecture due to diversity of their forms, glossy green leaves, abundant flowers, and attractive fruits. Many *Cotoneaster* shrubs vary in the plant form from the massive, erect *C. bullatus* that grows up to 5 m tall, to regular *C. nanshan*, to low, ground-hugging prostrate plants such as *C. horizontalis,* with twigs in a distinctive fish-bone pattern, to the ground cover *C. dammeri* (Figure 1). *Cotoneaster* leaves are alternate on the twigs, usually arranged in two rows, dark green on the top, with fine hairs underneath; the margin of the leaf blade is entire. The flowers are white or slightly pink, solitary or densely clustered at the branch tips. The morphological structure of flowers forms the basis for the classification of *Cotoneaster* members into two subgenera: *Cotoneaster*, in which pink or red flowers open successively over an extended period and the petals are erect (Figure 2a), and *Chaenopetalum*, in which mostly white flowers open simultaneously, with spreading petals (Figure 2b). The fruits are orange to red or black pomes, with one to five seeds, and ripen in September to October. In the autumn, the leaf and fruit colour draws attention, with dark green leaves and bright red fruits giving a showy blend of orange and red, which is a desirable decorative value of *Cotoneaster* species [3,4].

In addition to its high ornamental value, the *Cotoneaster* genus is a valuable source of plant materials, recommended in the traditional medicine of Iran, Turkey, Mongolia, and Tibet for the treatment of nasal haemorrhage, excessive menstruation, haemorrhoids, cardiovascular disorders, diabetes mellitus, neonatal jaundice, fever, and cough [5,6,7]. Phytochemical investigations into *Cotoneaster* species resulted in the identification of flavonoids, proanthocyanidins, phenolic acids, cyanogenic glycosides, triterpenoids, and phytoalexins, forming unique and diversified profiles in particular plant organs. The first phytochemical study of *Cotoneaster* plants can be traced back to 1974, when isoflavone compounds (biochanin A and its 7-*O*-glucoside) were isolated from the fruits and flowers of *C. serotina* and *C. pannosa* [8]. In a subsequent investigation on the leafy twigs of *C. oligantha*, (+)-catechin and (-)-epicatechin were isolated, as well as three isomeric B-type proanthocyanidins, with C4–C8 (or C4–C6) binding between flavan-3-ol monomers [9]. These studies initiated a number of further analyses leading to the isolation and identification of about 100 compounds, among which the flavonoids and proanthocyanidins are the most abundant phytochemicals in the genus *Cotoneaster*. These phenolics and *Cotoneaster* dry extracts of different polarity are also of interest in various biological studies, indicating their significant antioxidant, anti-inflammatory, hepatoprotective, anti-diabetic, anti-dyslipidaemic, anti-icteric, cardiotonic, antimicrobial, and other activities.

In the 2000s, two teams of researchers provided reviews of the chemical composition and biological activity of the genus *Cotoneaster*, but these concise studies are limited to only two species, *C. racemiflorus* [10] and *C. microphyllus* [11]. Additionally, a large number of scientific studies on this genus have emerged in recent years. To provide researchers with current information on the chemical composition and biological activity of the *Cotoneaster* species and to make a modest contribution to the rational therapeutic application of this genus, a systematic review of the genus *Cotoneaster* was imperative. I have covered these topics, including the phytochemistry, biological activity, and toxicology of the genus *Cotoneaster* through a literature survey from 1974 to early 2020.

## 2. Phytochemical Composition of the Genus *Cotoneaster*

### 2.1. Flavonoids and Proanthocyanidins

**Figure 3:**
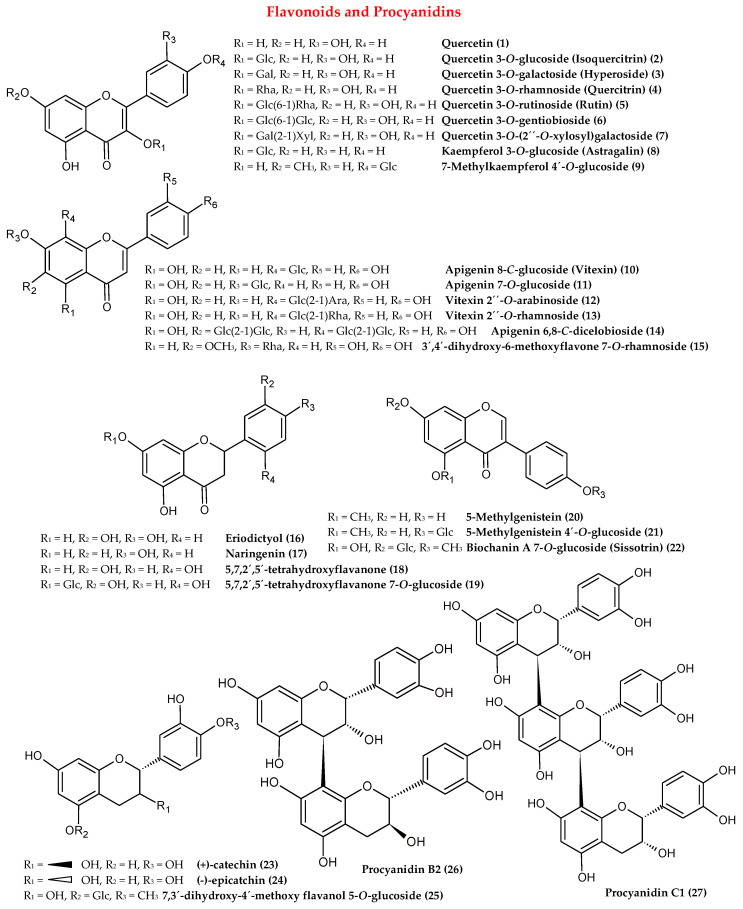
The chemical structures of flavonoids and procyanidins isolated from the genus *Cotoneaster.*

As the major bioactive constituents of *Cotoneaster* species are represented by flavonoids, most of the literature reports are focused on their identification and quantification. Numerous isolation studies have been carried out [8,12,13,14,15,16,17,18] to explore the rich composition of flavonoids found in the leaves, leafy twigs, flowers, fruits, and roots of several *Cotoneaster* species. As presented in Table 1, and Figure 3, the structurally diverse flavonoids are derivatives of flavonol (**1–9**), flavone (**10–15**), flavanone (**16–19**), flavanol (**23–25**), and isoflavone (**20–22**). The leaves, flowers, and fruits were found to be particularly rich in flavonols, mainly kaempferol and quercetin mono- and diglycosides, containing glucose, galactose, and rhamnose as the sugar moiety [17,18,19,20,21,22,23,24,25]. Regarding individual components, isoquercitrin (**2**), hyperoside (**3**), quercitrin (**4**), and rutin (**5**) were identified as the dominant flavonoids of the leaves, flowers, and fruits (Table 1). The content of flavonoids determined in the leaves of 12 selected *Cotoneaster* species by HPLC-PDA, after acid hydrolysis, as a sum of individual aglycones, was in the range 2.9–14.0 mg/g dw, with the highest level noted for *C. integerrimus*. In all leaf samples, quercetin was the most abundant aglycone with a level (2.6–13.2 mg/g dw) constituting 68–100% of total flavonoids quantified [19]. In the case of the bark and leafy twig samples, the representatives of flavanones and flavones, such as the eriodictyol hexosides (**16**) and *O*- and *C*-glycosides of apigenin (**10–14**), were the most characteristic flavonoids [7,15,22,24,26]. Considering the relatively rare occurrence of some flavonoids in nature, it is worth noting the presence of apigenin 6,8-*C*-dicellobioside (**14**) in the leafy twigs of *C. orbicularis*, as well as 5-methylgenistein 4′-glucoside (**21**) and biochanin A 7-*O*-glycoside (**22**) as the isoflavone representatives in the leafy twigs of *C. simonsii*, leaves of *C. mongolica*, fruits and flowers of *C. serotina* and *C. pannosa* [8,14,18].

(-)-Epicatechin (**24**) and (+)-catechin (**23**), as markers of proanthocyanidin occurrence, have been detected in several *Cotoneaster* species, suggesting the presence of procyanidins in this genus. Although the available literature on this phenolic group is limited to a few reports [9,17,19,20,21,22] and requires more extensive research, it is known that B-type procyanidins are characteristic of the *Cotoneaster* species, mostly present in the form of di-, tri-, and tetramers of (-)-epicatechin, and that (-)-epicatechin (**24**), procyanidins B2 (**26**), and C1 (**27**) are the dominant components of the leaves and fruits.

The polyphenols, with the largest contribution of flavonoids and procyanidins, have been quantified by HPLC-PDA and Folin-Ciocalteu (FC) methods in the leaves, fruits, and leafy twigs of various *Cotoneaster* species. As shown in Table 1, the highest polyphenol content was recorded for leaf samples (FC: 51.7–154.3 mg gallic acid equivalents (GAE)/g of leaf dry weight), especially for the leaves of *C. bullatus* and *C. zabelii* (FC: 154.3 and 124.9 mg GAE/g). These leaf samples were classified as polyphenol-rich plant material, with a level comparable to or even higher than those observed for other polyphenol abundant Rosaceae leaves, i.e., *Rosa canina*, *Aronia melanocarpa* and *Ribes nigrum* [19]. Due to the desired therapeutic value, this phenolic content might be further increased in dry methanol-water (7:3, *v/v*) extracts obtained from *Cotoneaster* leaves, reaching a level above 300 mg GAE/g of extract dry weight (Table 1) [20]. In other *Cotoneaster* plant parts, the level of phenolics is much lower, and maximally reached 113.6 and 49.5 mg GAE/g in the respective bark and fruits of *C. integerrimus* [22].

### 2.2. Phenolic Acids

**Figure 4:**
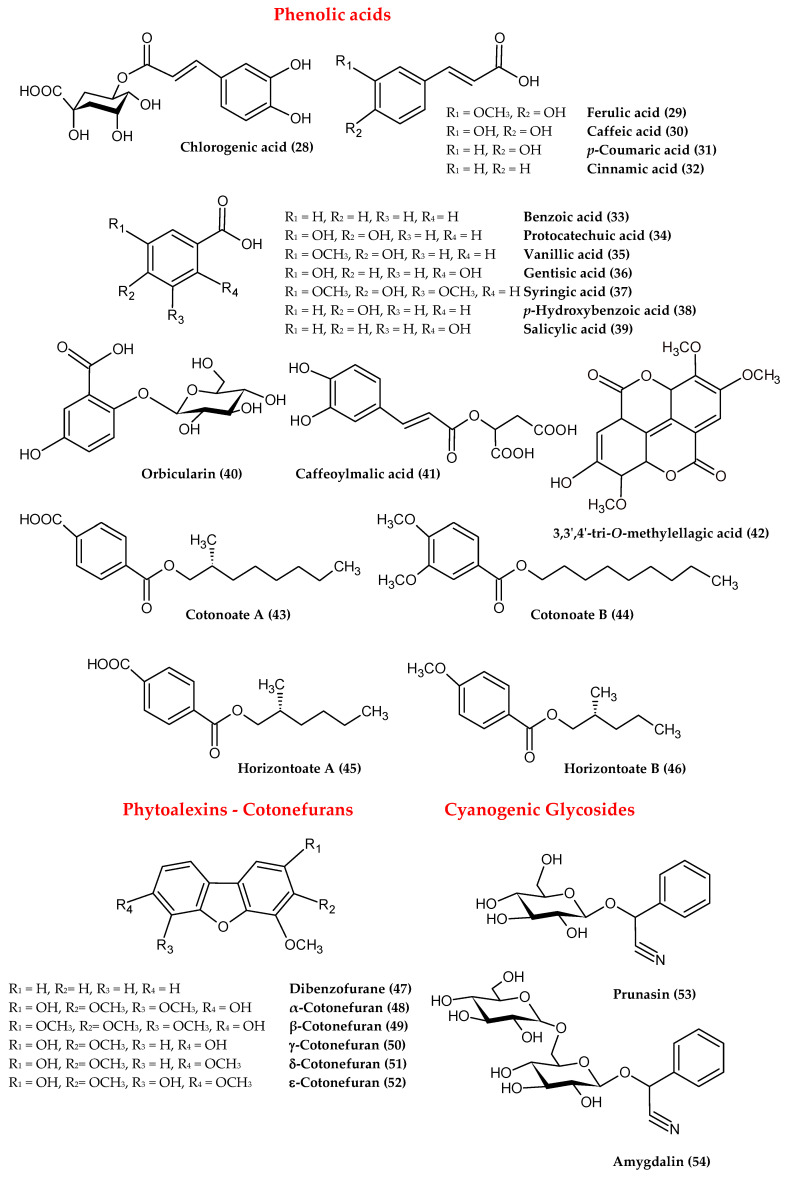

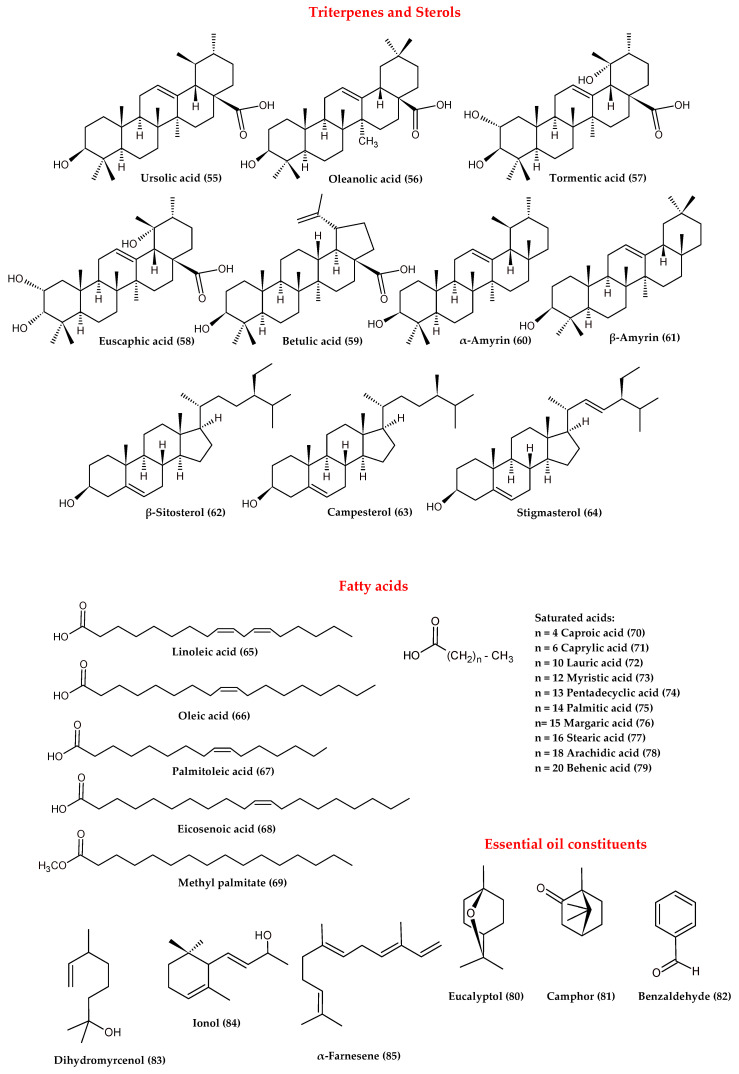

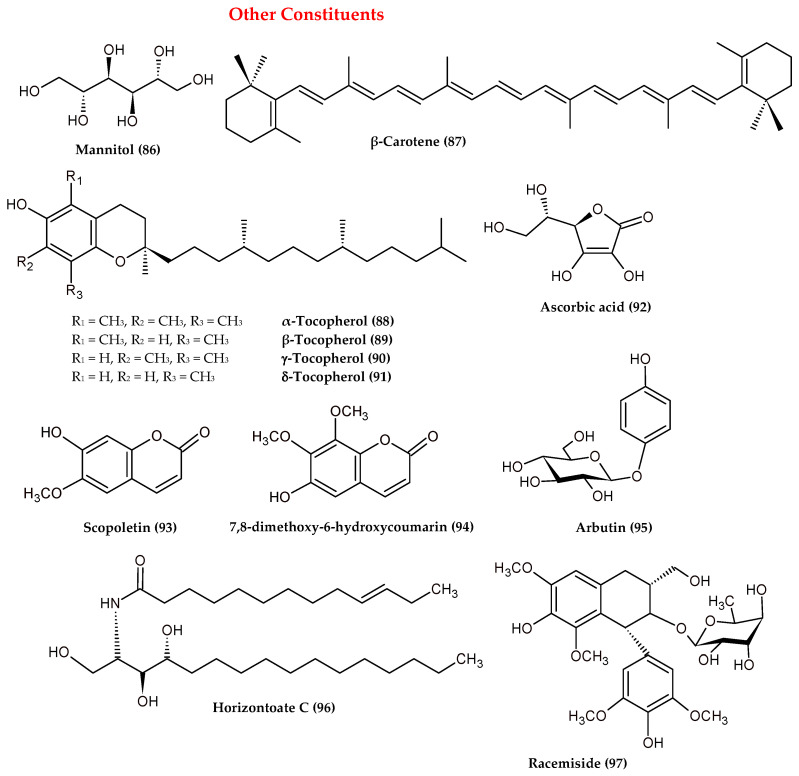
The chemical structures of the other compounds identified in the genus *Cotoneaster*.

Two different groups of phenolic acids (Figure 4), including *trans*-cinnamic and benzoic acids derivatives, were identified in the genus *Cotoneaster*. The typical representatives of these phenolics are caffeoylquinic acids such as chlorogenic acid (**28**) and its isomers, ferulic (**29**), caffeic (**30**), *p*-coumaric (**31**), cinnamic acids (**32**) as well as benzoic (**33**), protocatechuic (**34**), vanillic (**35**), gentisic (**36**), syringic (**37**), *p*-hydroxybenzoic (**38**), and salicylic acids (**39**) [5,7,13,15,20,21,23,26]. In addition, a gentisic acid 2-*O*-glucoside named orbicularin (**40**) was isolated from the leafy twigs of *C. orbicularis*, while caffeoylmalic acid (**41**) was obtained from the leaves of *C. zabelii* [15,17]. Nevertheless, in a set of identified phenolic acids, chlorogenic (**28**) and ferulic (**29**) acids were noted as the most abundant phenolics in the twigs and fruits of *C. integerrimus* [24], the leaves of *C. meyeri* [23], the leafy twigs of *C. nummularia* [7], and the leafy twigs of *C. horizontalis* [26]. Apart from typical phenolic acids, lesser-known compounds, including esters of benzoic and terephthalic acids, named cotonoates A (**43**), B (**44**), and horizontoates A (**45**), B (**46**), were isolated from the leafy twigs of *C. racemiflora* and *C. horizontalis*, respectively [27,28].

### 2.3. Phytoalexins

*Cotoneaster* plants are able to biosynthesise phytoalexins *de novo*, i.e., protective substances produced in response to infection by the pathogen or elicitation by abiotic agents such as a high concentration of heavy metals, thermal shock, and UV radiation [29]. In the case of the genus *Cotoneaster*, phytoalexins named cotonefurans have a dibenzofuran structure (**47**), with numerous hydroxyl and methoxyl substituents (Figure 4). The first phytoalexin of this type, named α-cotonefuran (**48**), was isolated in 1984 from *C. lactea* wood infected by the fungus *Ceratocystis ulmi* [30]. In further studies, four subsequent cononefurans, isolated from *C. acutifolius* wood after infection by *Nectria cinnabarina*, were marked with the symbols β, γ, δ, and ε (**49–52**) [29,31,32]. The anti-fungal ability of isolated cotoneafurans was confirmed in in vitro tests, demonstrating their significant activity against parasitic fungi such as *Alternaria alternata*, *Botrytis cinerea,* and *Fusarium culmorum* [31].

### 2.4. Cyanogenic Glycosides

Cyanogenic glycosides are crucial phytochemicals that may define the potential toxicity of *Cotoneaster* plants. The toxicity of cyanogenic glycosides is a result of their enzymatic degradation leading to the release of hydrogen cyanide (HCN), which, due to its affinity for Fe^3+^ ions, strongly inhibits the cytochrome oxidase enzyme system and blocks oxygen transfer from oxyhemoglobin to tissues [33]. In the genus *Cotoneaster*, belonging to the Rosaceae family well-known for the presence of cyanogenic glycosides, the occurrence of this group of compounds was also demonstrated (Figure 4). *Cotoneaster* fruits and leaves were found to contain mainly prunasin (**53**), the content of which, determined by the GC method in ethanolic leaf extracts, was in the range 0.5–5.0%, with the highest levels observed for *C. congesta* (5.0%), *C. praecox* (4.6%), and *C. integerrimus* (4.1%). In the fruits, prunasin (**53**) was accompanied by amygdalin (**54**), but its content did not exceed 0.1% in the studied extracts [34]. Amygdalin (**54**) was also found in the leafy twigs of *C. horizontalis*, and as demonstrated by HPLC, its level was low and safe with respect to health (0.334 mg/100 g of dry plant material) [35]. The toxicity of *Cotoneaster* fruits was then evaluated in in vivo studies conducted on a group of rats, administered by *C. divaricatus* fruits at doses of 0.5, 1.0, 2.0, and 4.0 g/kg body weight. The fruits at a dose of 0.5 g/kg were completely safe for the rats; the first symptoms of cyanide toxicity were observed at a dose of 1 g/kg [36].

### 2.5. Triterpenes, Sterols, and Fatty Acids

Additionally, some other compounds such as triterpenoids and sterols have been identified in the genus *Cotoneaster* (Figure 4). Among the most abundant triterpenes, ursolic acid (**55**) was isolated from the leaves and fruits of *C. serotina*, the fruits of *C. pannosa* [8], the leafy twigs of *C. simonsii* [14], the fruits of *C. microphylla* [37], the leafy twigs of *C. racemiflora* [38], the leaves of *C. mongolica* [18], and the leaves and twigs of *C. melanocarpus* [5]. In addition, four other triterpenes, including tormentic (**57**) and euscaphic (**58**) acids, were found in the leafy twigs of *C. simonsii* [14], as was betulic acid (**59**) in the fruits of *C microphylla* [37], and α-amyrin (**60**) in the leafy twigs of *C. horizontalis* [26]. Regarding the phytosterol profile, the presence of β-sitosterol (**62**) and stigmasterol (**64**) was noted in the leafy twigs of *C. horizontalis* [26]; β-sitosterol (**62**) and its 3-*O*-glucoside were found in the twigs of *C racemiflora* [38]. Based on gas chromatography-mass spectrometry (GC-MS) analysis, the fruits of nine *Cotoneaster* species were proved to contain four triterpenes (α- and β-amyrins (**60**, **61**), ursolic and oleanolic acids (**55**, **56**)) and three phytosterols (β-sitosterol, campesterol, and stigmasterol (**62–64**)). The total content of triterpenoids and sterols, depending on the tested species, was in the range of 154.6–515.6 mg/100 g of dry fruits, with highest level observed for *C. splendens* (515.6 mg/100 g) and *C. nanshan* (428.0 mg/100 g). The dominant compound in all fruit samples was β-sitosterol (**62**), with the highest content noted for *C. splendens* (463.3 mg/100 g), followed by *C. nanshan* (391.3 mg/100 g) and *C. horizontalis* (316.3 mg/100 g) [21].

Among other lipophilic compounds, fatty acids have also been found in the genus *Cotoneaster* (Figure 4). The GC-MS analysis of the fruits, seeds, and leafy twigs led to the identification of 15 compounds, including saturated, mono-, and polyunsaturated acids (**65–79**) with chain lengths ranging from 6 to 22 carbon atoms [25,26,39]. The content of fatty acids, assessed by GC-FID-MS in the fruits of nine *Cotoneaster* species, varied from 902.5 to 2683.8 mg/100 g of dry fruits, with the highest levels noted for *C. zabelii* (2683.8 mg/100 g) and *C. splendens* (2024.1 mg/100 g). In each fruit sample, linoleic acid (**65**) was recorded as the major fatty acid component with the highest level (above 10 mg/g) observed in the fruits of *C. zabelii*, *C. splendens*, *C. hjelmqvistii,* and *C. horizontalis* [21].

### 2.6. Essential Oil

The composition of the essential oil obtained by the standard hydrodistillation method was studied only for the leaves of *C. nummularoides* [40]. As demonstrated by GC-MS analysis, this volatile oil was dominated by mono- and bicyclic monoterpenes (Figure 4), among which eucalyptol (**80**; 24.7%), and camphor (**81**; 21.0%) were the most abundant ingredients [40]. One more report is available on the fragrance fraction isolated from leafy twigs of *C. horizontalis* by *n*-hexane-ethyl acetate extraction. Among the volatile components identified by GC-MS, benzaldehyde (**82**; 34.0%) and methyl palmitate (**69**; 10.0%) were predominant. The content of mono- and sesquiterpenes was at least twofold lower; dihydromyrcenol (**83**), 4.4%; ionol (**84**), 3.6%; and α-farnesene (**85**), 0.7% [26].

### 2.7. Carbohydrates

The species *C. tricolor*, *C. discolor*, *C. nummularius,* and *C. nummularioides* are valuable sources of manna, i.e., a thickened juice produced by the young shoots, in response to plant damage caused by parasitic insects. White or yellow manna pellets have cooling properties and a sweet taste, due to the presence of a rich polysaccharide fraction with predominantly mannitol (**86**) (up to 80% of total carbohydrates), followed by fructose, sucrose, and maltitol [41,42]. In another study, the aim of which was to assess the hypoglycaemic and anti-dyslipidaemic activity of the mucilage fraction obtained from the leafy twigs of *C. horizontalis*, a preliminary GC analysis of mucilage hydrolysate revealed a rich fraction of polysaccharides with predominantly glucose (46.2%), followed by xylose, arabinose, rhamnose, sorbitol, mannitol, ribose, fructose, and galacturonic acid [26].

### 2.8. Other Constituents

Among the other constituents, carotenoids, tocopherols, and vitamin C (Figure 4) have often been quantified in the fruits and leafy twigs of various *Cotoneaster* species. In the fruits of six *Cotoneaster* taxa, the level of carotenoids, determined by the spectrophotometric method, varied in the range of 0.19–0.55 mg/g of dry fruits, with the highest values (above 0.40 mg/g) found for *C. nebrodensis*, *C. mupensis*, *C. dielsianus,* and *C. melanocarpa* [43]. According to the HPLC-PDA results of individual compounds, β-carotene (**87**) was present at a concentration of 25 µg/g in the leafy twigs of *C. horizontalis*, while in the fruits of *C. pannosus* it was found at 14 µg/g [25,35]. The level of tocopherols, measured by HPLC-PDA in the petroleum ether extracts of *C. bullatus*, *C. dielsianus*, *C. francheti*, *C. moupinensis,* and *C. simonsii* seeds, varied in the range of 0.35–1.57 mg/g of dry extract, with the highest values noted for *C. simonsii* and *C. bullatus*. Among five individuals, γ-tocopherol (**90**) was characterised with the highest content, especially in *C. bullatus* seeds (2.86 mg/g of extract). The other α-, β-, and δ-tocopherols (**88**, **89**, **91**) were detected at levels at least three times lower (0.007–0.75 mg/g) [39]. Ascorbic acid (**92**), quantified by HPLC in the fruits of *C. pannosus*, was found at a level of 0.30 mg/g of dry fruits [25].

Among the other polyphenols, coumarins such as scopoletin (**93**) and 7,8-dimethoxy- 6-hydroxycoumarin (**94**) were isolated from the leafy twigs of *C. racemiflora* [38,44], while arbutin (**95**) was found in the leaves and twigs of *C. simonsii* [14]. Additionally, from the group of sphingolipids, an aliphatic amide called horizontoate C (**96**) was isolated from the leafy twigs of *C. horizontalis* [28]. The compound racemiside (**97**), as a representative of lignans, was also found in the leafy twigs of *C. racemiflora* [44].

## 3. Biological Activities of the Genus *Cotoneaster*

### 3.1. Traditional Usages

*Cotoneaster* species, as the most widespread plants in East Asia, are also natural medicines with a long traditional use in Iran, Pakistan, Turkey, Mongolia, and Tibet [5,27,45,46]. Numerous species are known to the local community for their diuretic, astringent, expectorant, hepatoprotective, digestive, cardiotonic, antiviral, and spasmolytic properties. Among the traditional indications for their use are inflammation of the eyes, wounds, bronchitis, fever, abdominal pain, pruritus, and leucoderma, as well as hemorrhoids, diabetes mellitus, and urinary calculi [7,35,38]. The *Cotoneaster* fruits are also used for culinary purposes as a valuable source of vitamins and minerals [24,37]. The fruits and twigs of *C. melanocarpus* are used in Mongolia and Nepal to treat nasal haemorrhage, excessive menstruation, and haematemesis [5]. In Turkey, the ripe fruits of *C. nummularia* are used as an expectorant, while in Iran, fruiting twigs of this species have a long traditional use as an antipyretic agent, applied for the relief of *Nobehfever* malarial fever [6,45]. In the Caucasus, the roots of *C. nummularia* are used to relieve rheumatic ailments [4]. Similar properties have been attributed to the leaves of *C. accuminata*, known for their external use in relieving rheumatoid arthritis and in the supportive treatment of scabies [16]. In turn, the roots of this species are an effective blood pressure-lowering agent [47]. The decoction of the *C. integerrimus* leaves and fruits is recommended as a strong antimicrobial agent for the treatment of digestive ailments, diabetes mellitus, fever, and cough [4,7,24]. In Iran, manna (locally called *Shri-e-Khesht*), i.e., the gum fraction obtained from the twigs of *C. tricolor* and *C. discolor*, orally administered to the mother or newborn baby, is considered an effective remedy for jaundice [48].

### 3.2. Pharmacological Activities

The *Cotoneaster* members, due to their wide traditional use in Asian medicine, have also attracted the interest of scientists, whose pharmacological investigations focused on the different polarity extracts and pure isolates, demonstrating their antioxidant, anti-inflammatory, antibacterial, antimalarial, hepatoprotective, anti-diabetic, anti-dyslipidaemic, and anti-jaundice properties.

#### 3.2.1. Antioxidant Activity

Among the numerous methods employed for the quantification of the antioxidant capacity of *Cotoneaster* species are the assays based on the single electron transfer (SET) and/or hydrogen atom transfer (HAT) reaction mechanisms. In vitro SET-based assays such as free radical scavenging with DPPH (2,2-diphenyl-1picrylhydrazyl radical), ABTS (2,2-azinobis (3-ethylbenzothiazoline-6-sulfonic acid) radical cation), DMPD (*N*, *N*-dimethyl-*p*-phenylenediamine radical cation), FRAP (ferric ion reducing antioxidant power), CUPRAC (cupric-reducing antioxidant capacity) as well as HAT-based methods for scavenging superoxide anions (O_2_^•−^), hydrogen peroxide (H_2_O_2_), and for the inhibition of linoleic acid peroxidation, are the most frequently methods used to assess the antioxidant properties of the leaves of 12 *Cotoneaster* species, including the most active *C. bullatus*, *C. zabelii* and *C. integerrimus* [17,19,20,22]; the leaves of *C. melanocarpus*, *C. meyeri*, *C. morulus*, *C. nummularia*, *C. mongolica* [5,18,23]; the fruits of nine *Cotoneaster* species [21] and the fruits of *C. pannosus* [25], the leafy twigs of *C. nummularia* and *C. afghanicus* [7,49]; the twigs, fruits of *C. integerrimus* [24] and sarcocarp of *C. multiflorus* [50]. As demonstrated in Table 2, in most applied tests, the *Cotoneaster* extracts displayed significant antioxidant effects, depending on the polarity of the extraction solvent. Particularly highly hydrophilic extracts showed antioxidant efficacy comparable to the results obtained for the industrial antioxidants, butylated hydroxyanisole (BHA), 2,6-di-*tert*-butyl-4-methylphenol (BHT), and Trolox. Considering that the antioxidant activity of *Cotoneaster* fruits and leaves is strongly dependent on the content of polyphenols, including flavonoids, procyanidins, and caffeoylquinic acids, these phytocompounds might be considered to play a significant role in their antioxidant capacity [19,20,22].

Among the individual polyphenols, isoquercitrin (**2**), hyperoside (**3**), quercitrin (**4**), rutin (**5**), quercetin 3-*O*-(2″-*O*-xylosyl)galactoside (**7**), (-)-epicatechin (**24**), procyanidins B2 (**26**) and C1 (**27**), chlorogenic acid (**28**), caffeoylmalic acid (**41**), isolated from the leaves of *C. bullatus* and *C. zabelii*, were also tested for their antioxidant activity (DPPH, FRAP, and inhibition of linoleic acid peroxidation assays). In comparison with the positive standards of Trolox and ascorbic acid, the highest antioxidant activity was obtained for the flavan-3-ol derivatives, including (-)-epicatchin (**24**) and procyanidins B2 (**26**), C1 (**27**) [17]. In the DPPH assay, significant antioxidant properties were also demonstrated for the polyphenols isolated from the leafy twigs of *C. racemiflora*, including 3,3′,4′-tri-*O*-methylellagic acid (**42**), scopoletin (**93**), 7,8-dimethoxy-6-hydroxycoumarin (**94**), and lignan racemiside (**97**) [44].

The antioxidant activity of the leaves of *C. bullatus*, *C. zabelii,* and *C. integerrimus* and the fruits of *C. zabelii*, *C. bullatus*, *C. splendens,* and *C. hjelmqvistii* was also proved in a more complex and in vivo–relevant model of human plasma exposed to oxidative stress induced by peroxynitrite (ONOO^−^). As demonstrated by the assessment of the total antioxidant status of plasma and the level of oxidative stress biomarkers (3-nitrotyrosine, free thiol groups, lipid hydroperoxides, and thiobarbituric acid-reactive substances), all *Cotoneaster* samples are able to normalise/enhance the nonenzymatic antioxidant capacity of human plasma and efficiently protect protein and lipid components of plasma against ONOO^−^-induced oxidative/nitrative damage [20,21]. This antioxidant effect may be responsible individual polyphenols such as isoquercitrin (**2**), hyperoside (**3**), quercitrin (**4**), rutin (**5**), quercetin 3-*O*-(2″-*O*-xylosyl)galactoside (**7**), (-)-epicatechin (**24**), procyanidins B2 (**26**) and C1 (**27**), chlorogenic acid isomers (**28**) and caffeoylmalic acid (**41**), for which antioxidant properties were also demonstrated in the biological model of human plasma exposed to oxidative/nitrogen stress induced by ONOO^−^[17]. *Cotoneaster* leaves and fruits were thus found to be a valuable source of antioxidants that might prevent against the negative effects of ONOO^−^. The harmful influence of ONOO^−^ is associated with serious pathological consequences in many organs and systems of the human body. In the case of the circulatory system, its harmful effect may result in a higher risk of cardiovascular disorders, such as stroke, myocardial infarction, or chronic heart failure [20]. Through the demonstrated protective effect of polyphenols on the nitration/oxidation of lipids and proteins in blood plasma, the use of *Cotoneaster* species might be regarded as a good strategy in the prophylaxis of various cardiovascular complaints.

#### 3.2.2. Anti-Inflammatory Activity

As shown in the literature, recently much focus has been devoted to screening novel drugs with anti-inflammatory potential isolated from various plant sources [51]. Anti-inflammatory activity has been reported for several *Cotoneaster* species [20,21,52]. In a recent comparative study of *Cotoneaster* leaves and fruits regarding their ability to inhibit lipoxygenase (LOX) and hyaluronidase (HYAL), all tested methanol-water (7:3) extracts were able to reduce the activity of both pro-inflammatory enzymes [20,21]. Among the leaf extracts, those obtained from *C. zabelii* and *C. bullatus* were found to be the most effective anti-inflammatory agents (IC_50_ = 217.8–185.8 µg/mL for LOX; and IC_50_ = 7.9–8.1 µg/mL for HYAL). Additionally, the inhibitory effect of these leaf extracts towards HYAL was higher or not statistically different than those of the positive controls, including indomethacin, an anti-inflammatory drug (IC_50_ = 8.6 µg/mL) [20]. In the case of nine *Cotoneaster* fruits, the strongest inhibitory effect towards LOX was demonstrated for the extracts of *C. hjelmqvistii* and *C. zabelli* (IC_50_ = 290.0 and 375.9 µg/mL), while the activity of HYAL was most strongly hindered by the extract of *C. lucidus* (IC_50_ = 25.7 µg/mL) [21]. In a recent study on *C. integerrimus* leaves, fruits, bark, and flowers, the methanol-water (7:3) extract from the bark was found to have the highest anti-inflammatory potential towards both LOX and HYAL (IC_50_ = 169.0 and 8.6 µg/mL, respectively) [22]. Further studies on this species conducted in a model of colon cancer (the HCT116 cell line) showed that the tested fruits and twigs had the ability to decrease the activity of key inflammatory cytokines involved in the pathophysiology of ulcerative colitis. All aqueous and methanolic extracts, tested at 100 µg/mL, significantly reduced H_2_O_2_-induced TNFα gene expression, while the methanolic extract of twigs was the most effective at inhibiting lipopolysaccharide (LPS)-induced production of nitrite, prostaglandin E2 (PGE2), and 8-iso-prostaglandin F2α (8-iso-PGF2α) [52].

#### 3.2.3. Antimicrobial Activity

Despite a report on the antiviral activity of *C. integrifolius* fruits, the data do not provide evidence that their methanolic extract shows significant activity against Herpes simplex virus type 1 (HSV-1) or influenza A virus. The antiviral activity was noted to be at least three times weaker (IC_50_ = 18.0 and 44.0 mg/mL, respectively) in comparison to the standards acyclovir (IC_50_ = 0.7 mg/mL, against the HSV-1) and amantadine hydrochloride (IC_50_ = 16.8 mg/mL, against influenza A) [46]. In a further study, the methanolic and aqueous extracts of *C. integrifolius* fruits and leafy twigs were also investigated for their antibacterial and antifungal activity against *Staphylococcus aureus*, methicillin-resistant *S. aureus* isolates (MRSA), *Escherichia coli*, *Pseudomonas aeruginosa*, *Klebsiella pneumoniae*, *Salmonella enteritidis*, *Streptococcus pneumoniae*, *Sarcina lutea*, as well as *Candida albicans* and *C. parasilopsis*. As a result, only the fruit methanolic extract exhibited significant antimicrobial activity, expressed as the value of the minimum inhibitory concentration (MIC = 0.195–6.25 mg/mL), especially noticeable against RMSA strains of *S. aureus* (MIC = 0.195–0.391 mg/mL). In addition, the aqueous and methanolic extracts from the fruits significantly inhibited the growth of *Candida albicans*, as evidenced by MIC = 0.391 mg/mL, that was comparable to the positive control gentamicin (MIC = 0.312 mg/mL) [24]. Using the diffusion-disk method, the antibacterial activity of the ethanolic extract of *C. acuminatus* roots (the concentration range: 1, 10, 50, and 100 µg/mL) was evaluated against *Bacillus subtilis*, *B. pumilus*, *Staphylococcus aureus*, *Microccocus glutamicus*, *Pseudomonas aeruginosa*, *Proteus vulgaris*, and *Escherichia coli*. The greatest effect was achieved for the extract concentration of 100 µg/mL, with growth inhibition zones (10–18 mm) comparable to those obtained with ampicillin (13–20 mm) [16]. Antibacterial effects were also assessed for methanolic extracts of *C. nummularioides* leaves (100, 200, 300, and 400 mg/mL) without significant activity, as evidenced by the values of the inhibition zone (6–12 mm) in relation to the control gentamicin (19–29 mm) [53].

#### 3.2.4. Antiparasitic Activity

The anti-malarial activity of the methanolic extract of *C. nummularia* fruit twigs was studied in vivo in a model of mice pre-infected with *Plasmodium berghei*. As a result, the oral administration of the extract at a concentration of 10 mg/kg in mice caused a moderate inhibition of *P. berghei* growth. This effect was assessed on the basis of the level of uninfected erythrocytes (41.9%) in comparison to the reference chloroquine, with which 100% healthy erythrocytes were obtained [6].

#### 3.2.5. Hepatoprotective, Anti-Diabetic, and Anti-Dyslipidaemic Effects

The hepatoprotective effect of the ethanolic extract of *C. horizontalis* leafy twigs was studied in vivo on rats with acetaminophen-induced liver damage (1 g/kg body weight). A positive effect was noted with the extract dose of 200 mg/kg body weight, as it effectively reduced the levels of liver dysfunction biomarkers, such as aspartate aminotransferase (AST), alanine aminotransferase (ALT), alkaline phosphatase (ALP), γ-glutamyl transferase (GGT), total bilirubin (TB), total proteins, and albumins (TP and TA). However, in comparison with the effects of the positive control silymarin (50 mg/kg body weight), the hepatoprotective activity of the tested extract was rather moderate [35]. Acetaminophen intoxication was also associated with rat hyperlipidemia and hypercholesterolemia, indicated by the measured values of triglycerides (TG) and total cholesterol (TC) as well as the low- and very low-density lipoproteins (LDL and VLDL). The acetaminophen-induced hypercholesterolemia might be attributed to decreased secretion of bile, bile salts, and total biliary fatty acids, while the increase in the triglyceride level may be a result of hepatic cell inhibition in the production of lipoproteins and lipases that are essential for triglyceride metabolism. According to the results, a significant, dose-dependent improvement in serum lipid profile parameters, similar to that obtained with silymarin, was clear evidence of the anti-dyslipidaemic activity of the *C. horizontalis* ethanolic extract [35]. In another in vivo study performed on rats, anti-diabetic and anti-dislipidaemic activity was investigated for the mucilage fraction of *C. horizontalis* leafy twigs, rich in monosaccharides, including glucose, xylose, arabinose, and rhamnose. As demonstrated in a 28-day trial with streptozotocin-induced diabetic rats, the mucus fraction (250 mg/kg body weight) was effective in the regulation of blood glucose levels and lipid distribution (TG, TC, LDL, and VLDL), with respect to glibenclamide (0.4 mg/kg body weight), a standard diabetes medication [26].

#### 3.2.6. Enzyme Inhibitory Activity

Several studies have been conducted to evaluate the ability of *Cotoneaster* species to inhibit the enzymes acetylcholinesterase (ACHE) and butyrylcholinesterase (BCHE) [7,23,24,28]. Inhibitors of these enzymes are used in the treatment of Alzheimer’s disease (AD), a progressive neurodegenerative disorder associated with memory impairment and cognitive deficit. A few literature reports have also documented the effectiveness of hydrophilic *Cotoneaster* extracts in the inhibition of tyrosinase, an skin pigmentation enzyme involved in the biosynthesis of melanin, as well as in the inhibition of α-amylase and α-glucosidase, which are digestive enzymes involved in the hydrolysis of polysaccharides [7,24].

The leaves of several *Cotoneaster*, *Amelanchier*, *Pyrus*, and *Sorbus* species were evaluated for their ability to inhibit ACHE and BCHE [23]. The genus *Cotoneaster* was represented by *C. meyeri*, *C. morulus*, and *C. nummularia*. Their leaf ethanolic extracts, studied at a concentration of 200 µg/mL, were able to inhibit ACHE and BCHE in the range of 10.6–35.1% and 10.6–50.7%, respectively. This efficacy was compared to that obtained with galantamine (100 µg/ mL; ACHE inhibition: 96.7; BCHE inhibition: 83.7%), a standard drug widely used in the treatment of Alzheimer’s disease. It is noteworthy that the activity of the *Cotoneaster* extract was comparable that of *Sorbus* leaves and higher than that for *Pyrus* samples [23].

The ethyl acetate, methanolic, and aqueous extracts of *C. nummularia* leafy twigs were also tested regarding the inhibition of ACHE and BCHE, as well as tyrosinase, α-amylase, and α-glucosidase [7]. The activity results were expressed as equivalents of standard inhibitors, i.e., galantamine (GALE) for ACHE and BCHE; kojic acid (KAE) for tyrosinase; and acarbose (ACAE) for α-amylase and α-glucosidase. According to the results, the tested extracts were able to inhibit both ACHE and BCHE (4.1–4.8 and 0.7–6.0 mg GALE/g extract, respectively), with the aqueous extract being more effective against ACHE (4.8 mg GALE/g), while the methanolic extract more active against BCHE (6.0 mg GALE/g). In addition, the methanolic extract was a more effective inhibitor of α-amylase and α-glucosidase (13.6 and 82.3 mg ACAE/g), while the aqueous extract was a tyrosinase inhibitor (32.3 mg KAE/g) [7]. The ability to inhibit ACHE, BCHA, tyrosinase, α-amylase, and α-glucosidase was also evaluated using the methanolic and aqueous extracts obtained from the fruits and leafy twigs of *C. integerrimus*. As demonstrated, the methanolic extracts were the most effective against ACHE, while the aqueous extracts against BCHE. Their effectiveness was comparable to or even greater than that of galantamine. With regard to tyrosinase, α-amylase, and α-glucosidase, it was found that most of the tested extracts were less effective than the corresponding kojic acid or acarbose standards [24].

Of the *Cotoneaster* individual compounds, only horizontoates A (**45**) and B (**46**), isolated from the leafy twigs of *C. horizontalis*, were evaluated in inhibitory tests of ACHE and BCHE, and their activity was comparable to those obtained for the standards galantamine and allanzanthane [28].

#### 3.2.7. Anti-Jaundice Activity

*Cotoneeaster* manna, a polysaccharide fraction used in traditional Asian medicine to treat neonatal jaundice, has also been of interest to Iranian scientists. Based on numerous clinical studies on neonates with jaundice [41,42,48,54,55,56], it was found that *C. tricolor* and *C. discolor* manna has the ability to regulate the excretion of bile from the liver and gallbladder. The serum bilirubin level was effectively reduced, with optimal effect achieved after 36 h of oral manna administration. As was suggested, mannitol has well-documented laxative properties and may be the main compound responsible for the anti-jaundice activity of manna. Due to the increased amount of bilirubin excreted with the faeces, its level in the blood decreased significantly, which contributed to relieving the jaundice symptoms. Based on these findings, *Cotoneaster* manna may be proposed as a traditional medicine to support standard phototherapy in the treatment of jaundice symptoms in newborns.

#### 3.2.8. Cytotoxic Activity

Despite a report on the cytotoxic activity of the leafy twigs of *C. horizontalis* [35], the data provide no evidence that this plant sample showed any real antitumor activity. The literature results refer only to moderate cytotoxic activity in various tumor cell lines (liver hepatocellular cells, HEPG2; human epithelial type 2 (HEp-2) cells; breast cancer cells, MCF7; cervical cells, HeLa, and HCT116 colon cancer cells), but not to animal experiments or human studies [35]. As is known, polyphenolic compounds, including those present in *Cotoneaster* extracts, bind to cells in culture, leading to the induction of a biological effect. Consequently, cytotoxicity or inhibition of proliferation is associated with changes in signaling pathways. Hence, this kind of over-interpretation of anti-tumor in vitro studies occurs very often, but has no pharmacological relevance.

Several in vitro studies have been conducted to assess the potential toxicity of *Cotoneaster* species against healthy cell lines. The ethanolic extract of *C. integrifolius* fruits was found to be non-toxic to Vero and MDCK cell lines, as evidenced by high CC_50_ values > 100 µg/mL [46]. No cytotoxic effect was observed for the methanolic extract of *C. nummularia* twigs (IC_50_ > 100 µg/mL) tested against the MDBK cell line, as assessed in comparison with the cytostatic compound 5-fluorouracil (IC_50_ = 0.3 µg/mL) [6]. Using an experimental model of peripheral blood mononuclear cells (PBMCs), no cytotoxic effect was found for the leaves of *C. zabelii*, *C. bullatus*, *C. integerrimus* and the fruits of *C. zabelii*, *C. bullatus*, *C. hjelmqvistii*, and *C. splendens* [20,21]. In a recent study, the ethanolic extract of *C. afghanicus* leafy twigs, tested at a concentration of 20 µg/180 µL of blood, was found to be safe, as only 4.0% hemolysis of erythrocytes was noted after their incubation in the presence of the examined extracts [49].

## 4. Methodology

The literature search was conducted using well-known databases (Scopus, Web of Science, ScienceDirect, and PubMed) using the keyword “*Cotoneaster*” with following the words: “composition”, “therapeutic”, “antioxidant”, “antimicrobial”, “anti-inflammatory”, “antiparasitic”, “hepatoprotective”, “anti-jaundice”, “cytotoxic” etc. Databases were extensively searched for original articles written in English, electronically published until July 2020. The validation of the articles was performed manually (by reading the entire article), and articles with a significant contribution to the field of research are included in the present review.

## 5. Conclusions

The present study is a literature survey of the *Cotoneaster* species that have been assessed regarding their phytochemistry, traditional uses, pharmacological activity, and toxicity. A total number of 30 *Cotoneaster* species are discussed with regard to the plant parts used and their bioactive lipophilic and hydrophilic compounds which are responsible for a wide range of pharmacological effects. The in vitro biological studies performed on the extracts of different polarity and purified isolates revealed a wide spectrum of biological activities of *Cotoneaster* species such as antioxidant, anti-inflammatory, antimicrobial, antiparasitic, and enzyme inhibitory effects. Based on the in vivo documented anti-jaundice, hepatoprotective, anti-diabetic, and anti-dyslipidaemic properties, it is justified to use the members of the *Cotoneaster* genus as auxiliary therapeutic agents, especially valuable in traditional medicine. I believe that the detailed information presented in this review on the phytochemistry and biological properties might provide an incentive for the proper evaluation of *Cotoneaster* use in traditional applications. In this regard, there is a need for further research on *Cotoneaster* extract standardisation and more detailed phytochemical studies. Considering pharmacological investigations, further in vivo studies on toxicity and bioactivity are required.

## Figures and Tables

**Table 1:** Flavonoids and procyanidins found in *Cotoneaster* species (presented on the basis of original studies published during the revised period 1974–2020; references in chronological order).

Species	Plant Part	Identified Compounds	Identification Method	Phenolic Content	Ref.
***C. serotina***	flowers	biochanin A 7-*O*-glucoside (sissotrin) (**22**)	isolation; UV, IR	-	[8]
***C. pannosa***	flowers, fruits	biochanin A 7-*O*-glucoside (sissotrin) (**22**)	isolation; UV, IR	-	[8]
***C. oligantha***	leafy twigs	isoquercitrin (**2**), quercetin 3-*O*-gentiobioside (**6**)	isolation; UV, IR, acid hydrolysis products	-	[12]
	leafy twigs	(+)-catechin (**23**), (-)-epicatechin (**24**), procyanidin dimer B type	isolation; UV, IR	-	[9]
***C. thymaefolia***	leaves	quercitrin (**4**), rutin (**5**), vitexin (**10**), vitexin 2″-*O*-arabinoside (**12**), vitexin 2″-*O*-rhamnoside (**13**); 5,7,2′,5′-tetrahyroxyflavanone (**18**), 5,7,2′,5′-tetrahyroxyflavanone 7-*O*-glucoside (**19**)	isolation; ^1^H, ^13^C NMR, FAB-MS, UV, acid hydrolysis products	-	[13]
***C. simonsii***	leafy twigs	7-methylkaempferol 4′-*O*-glucoside (**9**), 5-methylgenistein (**20**), 5-methylgenistein 4′-*O*-glucoside (**21**), (+)-catechin (**23**)	isolation; ^1^H, ^13^C NMR, DEPT, COSY, NOESY, FAB-MS, UV, acid hydrolysis products	-	[14]
***C. orbicularis***	leafy twigs	isoquercitrin (**2**), hyperoside (**3**), rutin (**5**), vitexin (**10**), vitexin 2″-*O*-rhamnoside (**13**), apigenin 6,8-*C*-dicellobioside (vicenin II 4″,4′′′-*O*-glucoside) (**14**), naringenin (**17**), (+)-catechin (**23**), (-)-epicatechin (**24**)	isolation; ^1^H, ^13^C NMR, HMBC, FAB-MS, UV, acid hydrolysis products	-	[15]
***C. horizontalis***	leafy twigs	luteolin, quercetin (**1**), naringenin (**17**), (+)-catechin (**23**)	HPLC-PDA	HPLC-PDA: 15.9 mg/g PMFC: 14.0 mg GAE/g ME	[26]
	leaves	isoquercitrin (**2**), hyperoside (**3**), quercitrin (**4**), rutin (**5**), quercetin 3-*O*-(2″-*O*-xylosyl)galactoside (**7**), quercetin 3-*O*-glucoside-7-*O*-rhamnoside, quercetin rhamnoside-hexoside, kaempferol rhamnoside-hexoside, quercetin dirhamnoside, (-)-epicatechin (**23**), procyanidins B2 (**26**) and C1 (**27**), procyanidin tetramer B-type	UHPLC-PDA-ESI-MS*^3^*	FC: 73.0 mg GAE/g PM	[19]
	fruits	isoquercitrin (**2**), hyperoside (**3**), quercitrin (**4**), rutin (**5**), quercetin 3-O-(2″-O-xylosyl)galactoside (**7**), quercetin rhamnoside-hexoside, (-)-epicatechin (**24**), procyanidins B2 (**26**) and C1 (**27**), procyanidins dimer and tetramer B-type	UHPLC-PDA-ESI-MS*^3^*	FC: 30.5 mg GAE/g PM	[21]
***C. acuminatus***	roots	apigenin 7-*O*-glucoside (**11**), 3′,4′-dihydroxy-6-methoxyflavone 7-*O*-rhamnoside (**15**), (-)-epicatechin (**24**), 7,3′-dihydroxy-4′-methoxyflavanol 5-*O*-glucoside (**25**)	isolation; ^1^H, ^13^C NMR, FAB-MS, IR, acid hydrolysis products	-	[16]
***C. melanocarpus***	leaves	isoquercitrin (**2**), hyperoside (**3**), rutin (**5**)	HPLC-PDA-MS	-	[5]
***C. nummularia***	leafy twigs	apigenin, luteolin, eriodictyol (**16**), (+)-catechin (**23**), (-)-epicatechin (**24**)	HPLC-PDA	HPLC-PDA: 58.6 mg/g MEFC: 251.8 mg GAE/g ME	[7]
***C. meyeri***	leaves	rutin (**5**)	HPLC-PDA	HPLC-PDA: 2.2 mg/g PM	[23]
***C. integerrimus***	twigs	quercetin (**1**), rutin (**5**), eriodictyol (**16**), apigenin, hesperidin, (-)-epicatechin (**24**)	HPLC-PDA	HPLC-PDA: 58.6 mg/g ME; FC: 115.1 mg GAE/g ME	[24]
	fruits	kaempferol, quercetin (**1**), rutin (**5**), (-)-epicatechin (**24**)	HPLC-PDA	HPLC-PDA: 20.8 mg/g MEFC: 38.5 mg GAE/g ME	[24]
***C. integerrimus***	fruits	kaempferol, hyperoside (**3**), rutin (**5**), (-)-epicatechin (**24**), procyanidins B2 (**26**) and C1 (**27**), procyanidins dimer and tetramer B-type	UHPLC-PDA-ESI-MS*^3^*	HPLC-PDA: 11.4 mg/g PMFC: 49.5 mg GAE/g PM	[22]
	leaves	quercetin (**1**), isoquercitrin (**2**), hyperoside (**3**), quercitrin (**4**), rutin (**5**), kaempferol hexoside, kaempferol rhamnoside-hexoside, quercetin rhamnoside-hexoside, (-)-epicatechin (**24**), procyanidins B2 (**26**) and C1 (**27**)	UHPLC-PDA-ESI-MS*^3^*	HPLC-PDA: 48.8 mg/g PMFC: 93.6 mg GAE/g PM HPLC-PDA: 139.0 mg/g ME FC: 241.3 mg GAE/g ME	[19,20,22]
	flowers	isoquercitrin (**2**), hyperoside (**3**), isorhamnetin hexoside, rutin (**5**), quercetin rhamnoside-hexoside, (+)-catechin (**23**), (-)-epicatechin (**24**), procyanidins B2 (**26**) and C1 (**27**), procyanidins dimer and tetramer B-type	UHPLC-PDA-ESI-MS*^3^*	HPLC-PDA: 36.0 mg/g PM FC: 107.0 mg GAE/g PM	[22]
	bark	isoquercitrin (**2**), hyperoside (**3**), myricetin hexoside, quercetin rhamnoside-hexoside, eriodictyol hexosides (**16**), (+)-catechin (**23**), (-)-epicatechin (**24**), procyanidins B2 (**26**) and C1 (**27**), procyanidins dimer and tetramer B-type	UHPLC-PDA-ESI-MS*^3^*	HPLC-PDA: 34.9 mg/g PM FC: 113.6 mg GAE/g PM	[22]
***C. bullatus***	leaves	isoquercitrin (**2**), hyperoside (**3**), quercitrin (**4**), rutin (**5**), quercetin 3-*O*-(2″-*O*-xylosyl)galactoside (**7**), quercetin rhamnoside-hexoside, quercetin dirhamnoside, (+)-catechin (**23**), (-)-epicatechin (**24**), procyanidins B2 (**26**) and C1 (**27**), procyanidins dimer, trimer and tetramer B-type	UHPLC-PDA-ESI-MS*^3^*	FC: 154.3 mg GAE/g PM HPLC-PDA: 84.7 mg/g ME FC: 332.9 mg GAE/g ME	[19,20]
	leaves	hyperoside (**3**), quercetin 3-*O*-(2″-*O*-xylosyl)galactoside (**7**)	isolation; ^1^H, ^13^C NMR, COSY, HMBC, HMQC, ESI-MS, acid hydrolysis products	-	[17]
	fruits	isoquercitrin (**2**), hyperoside (**3**), quercitrin (**4**), quercetin 3-*O*-(2″-*O*-xylosyl)galactoside (**7**), quercetin rhamnoside-hexoside, (-)-epicatechin (**24**), procyanidins B2 (**26**) and C1 (**27**), procyanidins dimer and tetramer B-type	UHPLC-PDA-ESI-MS*^3^*	FC: 37.3 mg GAE/g PM	[21]
***C. zabelii***	leaves	quercetin (**1**), isoquercitrin (**2**), hyperoside (**3**), quercitrin (**4**), rutin (**5**), quercetin 3-*O*-glucoside-7-*O*-rhamnoside, quercetin rhamnoside-hexoside, kaempferol rhamnoside-hexoside, quercetin dirhamnoside, (+)-catechin (**23**), (-)-epicatechin (**24**), procyanidins B2 (**26**) and C1 (**27**), procyanidins dimer, trimer and tetramer B-type	UHPLC-PDA-ESI-MS^3^	FC: 129.4 mg GAE/g PM HPLC-PDA: 69.7 mg/g ME FC: 311.5 mg GAE/g ME	[19,20]
	leaves	hyperoside (**3**), quercitrin (**4**), (-)-epicatechin (**24**), procyanidin B2 (**26**)	isolation; ^1^H, ^13^C NMR, COSY, HMBC, HMQC, ESI-MS, acid hydrolysis products	-	[17]
	fruits	isoquercitrin (**2**), hyperoside (**3**), rutin (**5**), quercetin rhamnoside-hexoside, (-)-epicatechin (**24**), procyanidins B2 (**26**) and C1 (**27**), procyanidins dimer and tetramer B-type	UHPLC-PDA-ESI-MS*^3^*	FC: 43.0 mg GAE/g PM	[21]
***C. tomentosus***	leaves	isoquercitrin (**2**), hyperoside (**3**), quercitrin (**4**), rutin (**5**), quercetin rhamnoside-hexoside, kaempferol rhamnoside-hexoside, quercetin dirhamnoside, (-)-epicatechin (**24**), procyanidins B2 (**26**) and C1 (**27**)	UHPLC-PDA-ESI-MS*^3^*	FC: 51.7 mg GAE/g PM	[19]
***C. melanocarpus***	leaves	isoquercitrin (**2**), hyperoside (**3**), quercitrin (**4**), rutin (**5**), kaempferol rhamnoside-hexoside, quercetin rhamnoside-hexoside, (-)-epicatechin (**24**), procyanidins B2 (**26**) and C1 (**27**)	UHPLC-PDA-ESI-MS*^3^*	FC: 54.8 mg GAE/g PM	[19]
***C. lucidus***	leaves	isoquercitrin (**2**), hyperoside (**3**), quercitrin (**4**), rutin (**5**), quercetin rhamnoside-hexoside, kaempferol rhamnoside-hexoside, (-)-epicatechin (**24**), procyanidins B2 (**26**) and C1 (**27**), procyanidins trimer and tetramer B-type	UHPLC-PDA-ESI-MS*^3^*	FC: 106.8 mg GAE/g PM	[19]
***C. lucidus***	fruits	isoquercitrin (**2**), hyperoside (**3**), rutin (**5**), quercetin rhamnoside-hexoside, (-)-epicatechin (**24**), procyanidins B2 (**26**) and C1 (**27**), procyanidin dimer B-type	UHPLC-PDA-ESI-MS*^3^*	FC: 28.7 mg GAE/g PM	[21]
***C. divaricatus***	leaves	isoquercitrin (**2**), hyperoside (**3**), quercitrin (**4**), rutin (**5**), quercetin rhamnoside-hexoside, kaempferol rhamnoside-hexoside, (-)-epicatechin (**24**), procyanidins B2 (**26**) and C1 (**27**), procyanidins dimer, trimer and tetramer B-type	UHPLC-PDA-ESI-MS*^3^*	FC: 119.7 mg GAE/g PM	[19]
	fruits	isoquercitrin (**2**), hyperoside (**3**), quercitrin (**4**), rutin (**5**), quercetin rhamnoside-hexoside, (-)-epicatechin (**24**), procyanidins B2 (**26**) and C1 (**27**), procyanidin dimer B-type	UHPLC-PDA-ESI-MS*^3^*	FC: 29.7 mg GAE/g PM	[21]
***C. nanshan***	leaves	isoquercitrin (**2**), hyperoside (**3**), rutin (**5**), quercetin 3-*O*-(2″-*O*-xylosyl)galactoside (**7**), quercetin rhamnoside-hexoside, (-)-epicatechin (**24**), procyanidins B2 (**26**) and C1 (**27**), procyanidin tetramer B-type	UHPLC-PDA-ESI-MS*^3^*	FC: 84.3 mg GAE/g PM	[19]
	fruits	isoquercitrin (**2**), hyperoside (**3**), rutin (**5**), (-)-epicatechin (**24**), procyanidin B2 (**26**), procyanidin dimer B-type	UHPLC-PDA-ESI-MS*^3^*	FC: 26.0 mg GAE/g PM	[21]
***C. hjelmqvistii***	leaves	isoquercitrin (**2**), hyperoside (**3**), quercitrin (**4**), rutin (**5**), quercetin 3-*O*-(2″-*O*-xylosyl)galactoside (**7**), quercetin rhamnoside-hexoside, kaempferol rhamnoside-hexoside, (-)-epicatechin (**24**),procyanidins B2 (**26**) and C1 (**27**), procyanidins dimer, trimer and tetramer B-type	UHPLC-PDA-ESI-MS*^3^*	FC: 124.9 mg GAE/g PM	[19]
	fruits	isoquercitrin (**2**), hyperoside (**3**), quercitrin (**4**), rutin (**5**), quercetin 3-*O*-(2″-*O*-xylosyl)galactoside (**7**), quercetin rhamnoside-hexoside, (-)-epicatechin (**24**), procyanidins B2 (**26**) and C1 (**27**), procyanidins dimer and tetramer B-type	UHPLC-PDA-ESI-MS*^3^*	FC: 43.5 mg GAE/g PM	[21]
***C. dielsianus***	leaves	isoquercitrin (**2**), hyperoside (**3**), quercitrin (**4**), rutin (**5**), quercetin 3-*O*-(2″-*O*-xylosyl)galactoside (**7**), quercetin rhamnoside-hexoside, kaempferol rhamnoside-hexoside, (-)-epicatechin (**24**), procyanidins B2 (**26**) and C1 (**27**), procyanidin tetramer B-type	UHPLC-PDA-ESI-MS*^3^*	FC: 69.9 mg GAE/g PM	[19]
	fruits	isoquercitrin (**2**), hyperoside (**3**), quercitrin (**4**), rutin (**5**), quercetin 3-*O*-(2″-*O*-xylosyl)galactoside (**7**), quercetin rhamnoside-hexoside, (-)-epicatechin (**24**), procyanidins B2 (**26**) and C1 (**27**), procyanidins dimer and tetramer B-type	UHPLC-PDA-ESI-MS*^3^*	FC: 31.0 mg GAE/g PM	[21]
***C. splendens***	leaves	isoquercitrin (**2**), hyperoside (**3**), quercitrin (**4**), rutin (**5**), quercetin 3-*O*-(2″-*O*-xylosyl)galactoside (**7**), quercetin rhamnoside-hexoside, kaempferol rhamnoside-hexoside, (-)-epicatechin (**24**), procyanidins B2 (**26**) and C1 (**27**), procyanidins dimer, trimer and tetramer B-type	UHPLC-PDA-ESI-MS*^3^*	FC: 99.2 mg GAE/g PM	[19]
	fruits	isoquercitrin (**2**), hyperoside (**3**), quercitrin (**4**), rutin (**5**), quercetin 3-O-(2″-O-xylosyl)galactoside (**7**), quercetin rhamnoside-hexoside, (-)-epicatechin (**24**), procyanidins B2 (**26**) and C1 (**27**), procyanidins dimer and tetramer B-type	UHPLC-PDA-ESI-MS*^3^*	FC: 38.5 mg GAE/g PM	[21]
***C. pannosus***	fruits	hyperoside (**3**), rutin (**5**), naringenin (**17**)	HPLC-PDA	HPLC-PDA: 4.0 mg/g PM	[25]
***C. mongolica***	leaves	quercetin (**1**), hyperoside (**3**), astragalin (**8**), biochanin A 7-*O*-glucoside (sissotrin) (**22**)	isolation; ^1^H, ^13^C NMR, acid hydrolysis products	-	[18]

The content of polyphenols was assessed by HPLC-PDA (high-performance liquid chromatography with diode array detector) or the Folin-Ciocalteu method (FC), and the results are expressed in gallic acid equivalents (GAE). The results were calculated based on the dry weight of the plant material (PM) or methanol-water (7:3, *v/v*) extract (ME). UHPLC-PDA-ESI-MS^*3*^—Ultra-performance liquid chromatography equipped with a photodiode array detector coupled to a mass spectrometer using the electrospray ionization interface; ^1^H, ^13^C NMR—Proton nuclear or carbon-13 nuclear magnetic resonances, respectively; COSY—Correlated spectroscopy; HMBC—Heteronuclear multiple bond correlation; HMQC—Heteronuclear multiple quantum correlation; DEPT—Distortionless enhancement by polarisation transfer; NOESY—Nuclear overhauser effect spectroscopy; IR—Infrared spectroscopy; FAB-MS, ESI-MS—Fast atom bombardment or electrospray ionization-mass spectrometry.

**Table 2:** Antioxidant activity reported for *Cotoneaster* species.

Species	Plant Part/Extract or Fraction	Antioxidant Assay	Antioxidant Effect	Ref.
***C. horizontalis***	leafy twigs/ethanolic extract	DPPH	EC_50_ = 19.3 µg/mL	[35]
***C. melanocarpus***	young and old twigs, leaves/ aqueous, methanolic, ethyl acetate, dichloromethane and hexane extracts	DPPH	aqueous extracts: EC_50_ = 53.5–86.7 µg/mL methanolic extracts: EC_50_ = 30.9–106.4 µg/mL ethyl acetate extracts: EC_50_ = 74.9–>200 µg/mL dichloromethane extracts: EC_50_ = 134.6–>200 µg/mL hexane extracts: EC_50_ = no activity	[5]
	leaves/methanol-water (7:3, *v/v*) extract	DPPH, O_2_^•−^, H_2_O_2_, FRAP	DPPH: EC_50_ = 32.75 µg/mL PM; O_2_^•−^: EC_50_ = 31.59 µg/mL PM H_2_O_2_: EC_50_ = 56.80 µg/mL PM; FRAP = 0.95 mmol Fe^2+^/g PM	[19]
***C. nummularia***	leafy twigs/aqueous, methanolic, ethyl acetate extracts	DPPH, ABTS, O_2_^•−^, FRAP, CUPRAC, phosphomolibdenum assay, β-carotene/linoleic acid assay, metal chelating assay	DPPH: EC_50_ = 0.097–0.252 mg/mL; ABTS: EC_50_ = 0.020–0.043 mg/mL; O_2_^•−^: EC_50_ = 1.066–1.603 mg/mL; FRAP = 0.143–0.654 mg/mL; CUPRAC: EC_50_ = 0.022–0.263 mg/mL phosphomolibdenum assay: EC_50_ = 56.1–177.3 mg AE/g β-carotene/linoleic acid assay: 85.5–93.0% (2 mg/mL; the extract concn.) metal chelating assay: EC_50_ = 0.3–18.7 mg EDTAE/g	[7]
	leaves/ethanolic extract	DMPD, metal chelating assay	DMPD: 20.9% (2 mg/mL; the extract concn.) metal chelating assay: 26.2% (2 mg/mL; the extract concn.)	[23]
***C. meyeri***	leaves/ethanolic extract	DMPD, metal chelating assay	DMPD: 8.2% (2 mg/mL; the extract concn.) metal chelating assay: 5.9% (2 mg/mL; the extract concn.)	[23]
***C. morulus***	leaves/ethanolic extract	DMPD, metal chelating assay	DMPD: 11.2% (2 mg/mL; the extract concn.) metal chelating assay: 21.5% (2 mg/mL; the extract concn.)	[23]
***C. integerrimus***	twigs/aqueous, methanolic extracts	DPPH, FRAP, CUPRAC, phosphomolibdenum assay, metal chelating assay	DPPH: EC_50_ = 1.06–1.09 mg/mL; FRAP = 257.4–265.4µg/mL; CUPRAC: EC_50_ = 336.5–349.0 µg/mL phosphomolibdenum assay: EC_50_ = 0.36–0.53 mg/mL metal chelating assay: EC_50_ = 1.47–6.24 mg/mL	[24]
	fruits/aqueous, methanolic extracts	DPPH, FRAP, CUPRAC, phosphomolibdenum assay, metal chelating assay	DPPH: EC_50_ = 1.85–2.44 mg/mL; FRAP = 376.4–498.8µg/mL; CUPRAC: EC_50_ = 596.2–657.4 µg/mL phosphomolibdenum assay: EC_50_ = 1.24–1.38 mg/mL metal chelating assay: EC_50_ = 2.14–6.14 mg/mL	[24]
	leaves/methanol-water (7:3) extract	DPPH, O_2_^•−^, H_2_O_2_, FRAP	DPPH: EC_50_ = 24.58 µg/mL PM; O_2_^•−^: EC_50_ = 29.49 µg/mL PM; H_2_O_2_: EC_50_ = 55.42 µg/mL PM; FRAP= 2.04 mmol Fe^2+^/g PM	[19]
***C. integerrimus***	leaves/methanol-water (7:3) extract	DPPH, FRAP, TBARS	DPPH: EC_50_ = 23.80 µg/mL PM; FRAP: 2.77 mmol Fe^2+^/g PM; TBARS: IC_50_ = 23.19 µg/mL PM	[22]
	leaves/methanol-water (7:3) extract and diethyl ether, ethyl acetate, *n*-butanol and water fractions prepared by fractionated extraction	DPPH, FRAP	DPPH: EC_50_ = 5.19–27.88 µg/mL; FRAP: 3.82–15.84 mmol Fe^2+^/g	[20]
	flowers/methanol-water (7:3) extract	DPPH, FRAP, TBARS	DPPH: EC_50_ = 20.65µg/mL PM; FRAP: 2.82 mmol Fe^2+^/g PM; TBARS: IC_50_ = 28.89 µg/mL PM	[22]
	bark/methanol-water (7:3) extract	DPPH, FRAP, TBARS	DPPH: EC_50_ = 20.76 µg/mL PM; FRAP: 2.75 mmol Fe^2+^/g PM; TBARS: IC_50_ = 26.01 µg/mL PM	[22]
	fruits/methanol-water (7:3) extract	DPPH, FRAP, TBARS	DPPH: EC_50_ = 54.17 µg/mL PM; FRAP: 1.10 mmol Fe^2+^/g PM; TBARS: IC_50_ = 47.32 µg/mL PM	[22]
***C. tomentosus***	leaves/methanol-water (7:3) extract	DPPH, O_2_^•−^, H_2_O_2_, FRAP	DPPH: EC_50_ = 34.50 µg/mL PM; O_2_^•−^: EC_50_ = 74.79 µg/mL PM; H_2_O_2_: EC_50_ = 91.30 µg/mL PM; FRAP= 0.90 mmol Fe^2+^/g PM	[19]
***C. lucidus***	leaves/methanol-water (7:3) extract	DPPH, O_2_^•−^, H_2_O_2_, FRAP	DPPH: EC_50_ = 25.35 µg/mL PM; O_2_^•−^: EC_50_ = 27.70 µg/mL PM; H_2_O_2_: EC_50_ = 38.46 µg/mL PM; FRAP= 2.18 mmol Fe^2+^/g PM	[19]
	fruits/methanol-water (7: 3) extract	DPPH, FRAP, TBARS	DPPH: EC_50_ = 123.41µg/mL PM; FRAP: 0.70 mmol Fe^2+^/g PM; TBARS: IC_50_ = 108.70 µg/mL PM	[21]
***C. divaricatus***	leaves/methanol-water (7:3) extract	DPPH, O_2_^•−^, H_2_O_2_, FRAP	DPPH: EC_50_ = 18.45 µg/mL PM; O_2_^•−^: EC_50_ = 37.57 µg/mL PM; H_2_O_2_: EC_50_ = 36.06 µg/mL PM; FRAP= 2.39 mmol Fe^2+^/g PM	[19]
	fruits/methanol-water (7:3) extract	DPPH, FRAP, TBARS	DPPH: EC_50_ = 91.47 µg/mL PM; FRAP: 0.76 mmol Fe^2+^/g PM; TBARS: IC_50_ = 83.16 µg/mL PM	[21]
***C. horizontalis***	leaves/methanol-water (7:3) extract	DPPH, O_2_^•−^, H_2_O_2_, FRAP	DPPH: EC_50_ = 23.02 µg/mL PM; O_2_^•−^: EC_50_ = 49.48 µg/mL PM; H_2_O_2_: EC_50_ = 71.14 µg/mL PM; FRAP= 2.08 mmol Fe^2+^/g PM	[19]
	fruits/methanol-water (7:3) extract	DPPH, FRAP, TBARS	DPPH: EC_50_ = 93.32 µg/mL PM; FRAP: 0.85 mmol Fe^2+^/g PM; TBARS: IC_50_ = 84.89 µg/mL PM	[21]
***C. nanshan***	leaves/methanol-water (7:3) extract	DPPH, O_2_^•−^, H_2_O_2_, FRAP	DPPH: EC_50_ = 24.68 µg/mL PM; O_2_^•−^: EC_50_ = 55.74 µg/mL PM; H_2_O_2_: EC_50_ = 54.17 µg/mL PM; FRAP= 1.70 mmol Fe^2+^/g PM	[19]
	fruits/methanol-water (7:3) extract	DPPH, FRAP, TBARS	DPPH: EC_50_ = 178.35 µg/mL PM; FRAP: 0.61 mmol Fe^2+^/g PM; TBARS: IC_50_ = 165.76 µg/mL PM	[21]
***C. hjelmqvistii***	leaves/methanol-water (7:3) extract	DPPH, O_2_^•−^, H_2_O_2_, FRAP	DPPH: EC_50_ = 21.04 µg/mL PM; O_2_^•−^: EC_50_ = 28.10 µg/mL PM; H_2_O_2_: EC_50_ = 35.57 µg/mL PM; FRAP= 2.59 mmol Fe^2+^/g PM	[19]
	fruits/methanol-water (7:3) extract	DPPH, FRAP, TBARS	DPPH: EC_50_ = 64.51 µg/mL PM; FRAP: 1.05 mmol Fe^2+^/g PM; TBARS: IC_50_ = 62.96 µg/mL PM	[21]
***C. dielsianus***	leaves/methanol-water (7:3) extract	DPPH, O_2_^•−^, H_2_O_2_, FRAP	DPPH: EC_50_ = 29.49 µg/mL PM; O_2_^•−^: EC_50_ = 54.50µg/mL PM; H_2_O_2_: EC_50_ = 62.97 µg/mL PM; FRAP= 1.71 mmol Fe^2+^/g PM	[19]
	fruits/methanol-water (7:3) extract	DPPH, FRAP, TBARS	DPPH: EC_50_ = 117.10 µg/mL PM; FRAP: 0.67 mmol Fe^2+^/g PM; TBARS: IC_50_ = 103.72 µg/mL PM	[21]
***C. splendens***	leaves/methanol-water (7:3) extract	DPPH, O_2_^•−^, H_2_O_2_, FRAP	DPPH: EC_50_ = 22.56 µg/mL PM; O_2_^•−^: EC_50_ = 44.07 µg/mL PM; H_2_O_2_: EC_50_ = 43.24 µg/mL PM; FRAP= 2.44 mmol Fe^2+^/g PM	[19]
	fruits/methanol-water (7:3) extract	DPPH, FRAP, TBARS	DPPH: EC_50_ = 67.15 µg/mL PM; FRAP: 0.98 mmol Fe^2+^/g PM; TBARS: IC_50_ = 66.21 µg/mL PM	[21]
***C. bullatus***	leaves/methanol-water (7:3) extract	DPPH, O_2_^•−^, H_2_O_2_, FRAP	DPPH: EC_50_ = 20.91 µg/mL PM; O_2_^•−^: EC_50_ = 37.80 µg/mL PM; H_2_O_2_: EC_50_ = 28.95 µg/mL PM; FRAP= 3.76 mmol Fe^2+^/g PM	[19]
	leaves/methanol-water (7:3) extract and diethyl ether, ethyl acetate, *n*-butanol and water fractions prepared by fractionated extraction	DPPH, FRAP	DPPH: EC_50_ = 3.19–22.41 µg/mL; FRAP: 3.99–16.99 mmol Fe^2+^/g	[20]
	fruits/methanol-water (7:3) extract	DPPH, FRAP, TBARS	DPPH: EC_50_ = 66.31 µg/mL PM; FRAP: 0.97 mmol Fe^2+^/g PM; TBARS: IC_50_ = 64.99 µg/mL PM	[21]
***C. zabelii***	leaves/methanol-water (7:3) extract	DPPH, O_2_^•−^, H_2_O_2_, FRAP	DPPH: EC_50_ = 21.52 µg/mL PM; O_2_^•−^: EC_50_ = 52.76 µg/mL PM; H_2_O_2_: EC_50_ = 43.28 µg/mL PM; FRAP= 2.98 mmol Fe^2+^/g PM	[19]
	leaves/methanol-water (7:3) extract and diethyl ether, ethyl acetate, *n*-butanol and water fractions prepared by fractionated extraction	DPPH, FRAP	DPPH: EC_50_ = 3.95–18.73 µg/mL; FRAP: 3.89–16.74 mmol Fe^2+^/g	[20]
	fruits/methanol-water (7:3) extract	DPPH, FRAP, TBARS	DPPH: EC_50_ = 62.93 µg/mL PM; FRAP: 1.09 mmol Fe^2+^/g PM; TBARS: IC_50_ = 62.54µg/mL PM	[21]
***C. mongolica***	leaves/methanolic extract and chloroform, *n*-butanol and water fractions prepared by fractionated extraction	DPPH	EC_50_ = 55.7–>200 µg/mL	[18]
	fruits/ methanolic extract and *n*-butanol fraction prepared by fractionated extraction	DPPH	EC_50_ = 108.5–>200 µg/mL	[18]
***C. pannosus***	fruits/ethanol:water (7:3), ethanol hexane (55:45) extracts	DPPH	EC_50_ = 47.3–54.9 µg/mL	[25]
***C. afghanicus***	leafy twigs/hexane, ethanolic extracts	DPPH	EC_50_ = 57.4–64.7 µg/mL	[49]

The results were calculated based on the dry weight of the extracts/fractions or the plant material (PM); EC_50_, IC_50_—The half-maximal effective or inhibitory concentration, respectively; DPPH—2,2-Diphenyl-1-picrylhydrazyl radical scavenging assay; O_2_^•−^—Superoxide anion radical assay; H_2_O_2_—Hydrogen peroxide scavenging assay; FRAP—Ferric reducing antioxidant assay; CUPRAC—Cupric-reducing antioxidant capacity; DMPD—*N*,*N*-Dimethyl-*p*-phenylenediamine assay; TBARS—Linoleic acid peroxidation assay with quantification of thiobarbituric acid-reactive substances.
